# Comparative Analysis of Angora Rabbit Colostrum and Mature Milk Using Quantitative Proteomics

**DOI:** 10.3390/biology13080634

**Published:** 2024-08-19

**Authors:** Dongwei Huang, Yuanlang Wang, Haisheng Ding, Huiling Zhao

**Affiliations:** Anhui Provincial Key Laboratory of Livestock and Poultry Product Safety Engineering, Institute of Animal Husbandry and Veterinary Medicine, Anhui Academy of Agricultural Sciences, Hefei 230031, China; huangdongwei@aaas.org.cn (D.H.); wangyuanlang@aaas.org.cn (Y.W.); dinghaisheng@aaas.org.cn (H.D.)

**Keywords:** rabbit milk, colostrum, mature milk, proteomic, DIA

## Abstract

**Simple Summary:**

Rabbit colostrum is the initial mammary secretion after parturition, consisting of nutritional and bioactive components. However, the protein expression profiles in the colostrum and mature milk of rabbits are still unclear. Accordingly, in this study, the proteomics approach was performed on milk sampled from rabbit colostrum and mature milk. A total of 525 differentially abundant proteins (DAPs) were revealed, among which 244 proteins were upregulated and 281 were downregulated compared with the colostrum of rabbits. The bioinformatics analysis showed that the DAPs were related to the immune response and fatty acids metabolism. In conclusion, the results of this study will contribute to the expansion of the rabbit milk protein database, as well as provide insights into utilizing breeding practices to address concerns related to the growth and health of rabbit offspring.

**Abstract:**

Colostrum intake is a crucial determinant of survival in newborn rabbits. Neonates rely entirely on passive immunity transfer from their mothers while suckling colostrum. The goal of this study was to explore the protein differences of rabbit milk during different lactation periods. Our findings showed that the daily milk yield exhibited an increasing trend from the 2nd to the 21st day of lactation. A data-independent acquisition proteomics approach identified a total of 2011 proteins. Significantly, different abundances were found for 525 proteins in the colostrum and the mature milk samples. Eleven differentially abundant proteins (DAPs) were examined using parallel reaction monitoring, which verified the reliability of the proteomic data. Gene Ontology analysis revealed that these DAPs were primarily associated with glycosyltransferase activity, macromolecule transmembrane transporter activity, and regulation of acute inflammatory response. The dominant metabolic pathways of the DAPs involve the complement and coagulation cascades. A protein–protein interaction analysis identified apolipoprotein B, apolipoprotein A1, triose phosphate isomerase 1, and albumin as the hub proteins responsible for distinguishing differences between biological properties in rabbit colostrum and mature milk. These findings enhance our comprehension of the rabbit milk proteome, particularly in expanding our knowledge regarding the requirements of neonatal rabbits.

## 1. Introduction

Milk is a natural fluid produced by the mammary glands of mammals and provides nutrition to newborns. Milk contains many nutrients, including proteins, lactose, fats, vitamins, and minerals [1]. These components are not constant but vary depending on factors such as species, breed, lactation period, and nutrition level [2]. Rabbit milk has special nutritional value and health care functions. Compared with cow and sow milk, rabbit milk is two and three times more concentrated in fat (12.7–18.9%) and protein (11.9–14.7%), respectively, and it contains one-third of the amount of lactose found in the other two milk types (1.0–1.9%) [3]. Moreover, it has an extremely high content of short-chain fatty acids, which play a pivotal role in protecting the gut and maintaining the health of kits [4].

Colostrum, the initial milk secreted by the mammary glands of mammals, is produced immediately after parturition. Colostrum proteins not only provide essential nutrition for the neonate but also participate in the early postnatal transfer of passive immunity. Additionally, colostrum contains enzymes that facilitate digestion and proteins involved in gastrointestinal tract regulation throughout a mammal’s lifespan [5,6]. In terms of composition, colostrum has a higher concentration of milk fat and protein than mature milk. Consequently, colostrum has a greater density and a more pronounced yellow hue than mature milk [7]. Immunoglobulins constitute the primary protein fraction of colostrum, encompassing a diverse array of unique proteins with distinct functional roles. These include neutrophil- and macrophage-secreted, blood complement system, and acute-phase proteins, as well as specific antimicrobial peptides and proteins [8,9]. While colostrum immunoglobulin content has traditionally been regarded as the primary determinant of colostrum quality, recent advancements in proteomics have revealed the dynamic nature of both colostrum and mature milk, wherein various cytokines, growth factors, or other bioactive compounds with relatively low concentrations may be present [10].

Rabbits are diminutive herbivorous creatures characterized by distinctive physiological traits, including a brief lactation period, elevated levels of fat and protein in the milk, and the rapid production of offspring [11]. The growth and survival of neonatal rabbits is entirely dependent on the provision of milk by the doe [3], with particular emphasis on ensuring an adequate intake of colostrum within the first 2–3 h after birth. This is crucial because colostrum production in neonatal rabbits occurs shortly after birth, and the mechanisms facilitating passive immunity transfer and colostrum absorption function for only a limited time [12]. However, research on rabbit colostrum remains limited with only a few studies reporting the chemical composition and morphological characteristics of both rabbit colostrum and mature milk [3]. Therefore, it is of great importance to uncover and compare the protein profiles of rabbit colostrum and mature milk, which may provide a better understanding of the protein composition and potential biological functions across lactation stages. Furthermore, the proteomes of colostrum and mature milk could offer valuable insights into dam health and performance during lactation initiation by facilitating the identification of rabbit milk protein biomarkers [13].

Over the past few years, advancements in proteomics, accompanied by the emergence of novel instrumentation, have addressed the challenge of identifying low-abundance proteins and facilitated a more rapid and comprehensive characterization of the proteome [1]. Numerous proteins have been successfully identified and quantified in colostrum and mature milk samples from various species, including cows [13,14], goats [15], and donkeys [16], but these factors have not been identified in rabbits. Data-independent acquisition (DIA) is a novel mass spectrometry data acquisition method developed in recent years that has the advantages of high throughput, high resolution, high reproducibility, and accurate quantification that has facilitated quantitative proteomic analysis [17]. This novel proteomic technique enables a more comprehensive characterization of rabbit milk at different lactation stages.

In this study, we employed a DIA proteomics analysis to identify protein components and investigate the protein differences between rabbit colostrum and mature milk. Our findings will contribute to the expansion of the rabbit milk protein database, elucidating the temporal dynamics in the rabbit milk proteome, and provide a theoretical foundation for optimizing and utilizing milk products.

## 2. Materials and Methods

### 2.1. Milk Sample Collection and Preparation

All procedures involving animal handling were approved by the Animal Care Advisory of Agricultural Sciences (AAAS2023-42) and the Guidelines for Experimental Animals of the Ministry of Science and Technology (Beijing, China). Raw milk samples were collected from five one-year-old healthy female Angora rabbits (*Oryctolagus cuniculus*), randomly selected from the Experimental Farm of the Anhui Academy of Agricultural Sciences, Anhui, China, on the second parity. The litter sizes (kits born alive) and milk yields of the five female Angora rabbits were recorded on days 1, 7, 14, 21, and 28 during lactation. Milk yields were calculated following the Peaker & Taylor method [18], with minor modifications to account for suckling durations of less than five minutes. Colostrum and mature milk were collected during the initial morning milking session after kindling within 2 h and 21 d, respectively. By gently massaging the mammary glands, the raw milk sample was obtained and placed in a cryotube. Overall, five of the samples were colostrum (2 h postpartum) and five were mature milk (21 d postpartum). Following collection, all raw milk samples were promptly frozen and transported to the laboratory for storage at −80 °C until analysis.

### 2.2. Total Protein Extraction

After thawing, the raw milk samples were centrifuged at 12,000× *g* and 4 °C for 10 min. The supernatant was then transferred to a new centrifuge tube. The 14 most abundant proteins were removed using a Pierce^TM^ Top 14 Abundant Protein Depletion Spin Column Kit (Thermo Scientific, Waltham, MA, USA). The signal intensity of medium- and low-abundance proteins was significantly enhanced, resulting in an increased number of identified proteins. Protein concentrations were determined using a BCA Protein Assay kit (Sigma, St. Louis, MO, USA), with bovine serum albumin as a standard, according to the manufacturer’s instructions.

### 2.3. Trypsin Digestion

Protein solutions were subjected to reduction with 5 mM of dithiothreitol (Bio-Rad, Hercules, CA, USA) at 56 °C for 30 min, followed by alkylation with 11 mM of iodoacetamide (Bio-Rad, Hercules, CA, USA) at room temperature in the dark for 15 min to facilitate digestion. Subsequently, each protein sample was diluted using 100 mM of TEAB buffer (Sigma, St. Louis, MO, USA) until the urea concentration was <2 M. In the initial digestion step, trypsin (Promega, Madison, WI, USA) was added at a trypsin-to-protein mass ratio of 1:50 and allowed to digest proteins overnight. A second round of digestion was performed at a trypsin-to-protein mass ratio of 1:100 for 4 h. Finally, the desalting of peptides was achieved using Strata X C18 SPE columns (Phenomenex, Torrance, CA, USA).

### 2.4. Data-Dependent Acquisition (DDA) and DIA Analyses

Dried tryptic peptides were dissolved in 0.1% (*v*/*v*) of formic acid (FA) and subjected to EASY-nLC 1200 ultra-performance liquid chromatography (UPLC) (Thermo Scientific, Waltham, MA, USA). The gradient was increased from solvent A (0.1% FA in 2% acetonitrile) to solvent B (0.1% FA in 90% acetonitrile) over 120 min at a constant flow rate of 450 nL/min. The peptides were subjected to nano spray ionization, followed by tandem mass spectrometry (MS/MS) using an Orbitrap Exploris^TM^ 480 mass spectrometer (Thermo Scientific, Waltham, MA, USA) with a nano-electrospray ion source. The electrospray voltage applied was 2.3 kV, and the high-field asymmetric waveform ion mobility spectrometry (FAIMS) compensation voltage applied was −70 V; −45 V. An Orbitrap Exploris^TM^ 480 equipped with a FAIMS interface significantly improves detection sensitivity and effectively reduces the effect of high-abundance protein on depth [19,20].

For the DDA analysis, MS was performed in positive ion mode with a parent ion scanning range of 300–1800 *m*/*z* and a mass resolution of 120,000, with the automatic gain control (AGC) target set at 3 × 10^6^. The 20 most abundant ions with a charge ≥ 2 from the MS scan were selected and fragmented by higher-energy collisional dissociation (HCD) with normalized collision energies of 27 eV. MS/MS. Spectra were obtained at a resolution of 30,000 with an AGC target of 2 × 10^5^ and a maximum injection time of 80 ms. The Q Exactive HF dynamic exclusion was set for 40.0 s and run in positive mode.

For the DIA analysis, MS was performed in the positive ion mode with a parent ion scanning range of 400–1200 *m*/*z* for a full scan in the primary MS, with the AGC target value set at 3 × 10^6^. The 32 acquisition windows in the MS were fragmented using HCD with a collision energy of 28 eV; each acquisition window was 26 *m*/*z*. MS/MS spectra were obtained at a resolution of 30,000 with an AGC target of 1 × 10^6^, and the maximum injection time was set to auto and run in positive mode.

### 2.5. Protein Identification and Quantification

The raw DIA files were analyzed using MaxQuant software (version 2.0.3.0) to search the UniProt database. The relevant parameters were set as follows: the digestion mode was set to Trypsin/P specificity; maximum missed cleavages was two; fixed carbamidomethyl modification of cysteine; and methionine oxidation. Protein and peptide identification was achieved at a false discovery rate (FDR) and peptide spectrum matching of 0.01. A spectral library was built using the DDA LC-MS/MS spectral data (.data format) in Skyline (version 4.1.0), with the DDA search results imported into Spectronaut Pulsar [21,22]. The DIA data were analyzed using Spectronaut to search the constructed spectral library. The main software parameters were set as follows: the retention time prediction type was dynamic iRT and interference with MS2 level correction and cross-run normalization were enabled. All results were filtered using a Q-value cutoff of 0.01. The quantitative results for proteins were statistically analyzed using ANOVA with the Benjamini–Hochberg FDR. Differentially abundant proteins (DAPs) were considered proteins with a |foldchange| > 1.5 and *p*-value < 0.05.

### 2.6. Bioinformatics and Statistical Analysis

A principal component analysis (PCA) of the quantified proteins was performed using the “FactoMineR” and “Factoextra” packages in R software (version 4.0.5). This visualization illustrates the relationship between milk samples obtained from the studied groups. The cellular component, molecular function and biological process of all DAPs according to their Gene Ontology (GO) annotations and Kyoto Encyclopedia of Genes and Genomes (KEGG) pathway analysis were achieved using DAVID Bioinformatics Resources 6.8 (https://david.ncifcrf.gov/, accessed on 24 June 2024). Protein–protein interactions (PPIs) of DAPs were predicted using the Search Tool for Retrieval of Interacting Genes (STRING) database (https://string-db.org, accessed on 25 June 2024) with 0.7 confidence and visualized using Cytoscape software (version 3.10.0). The CytoHubba application in Cytoscape was used to screen the hub proteins.

### 2.7. Parallel Reaction Monitoring (PRM) Validation

PRM was used to verify the relative expression levels of the DAPs obtained from the DIA analysis. Milk samples collected from each Angora rabbit in the first 2 h and 3 d after kindling were confirmed by PRM using an acquired MS/MS spectrum. The proteins extracted from each sample were quantitatively and qualitatively assessed, enzymatically hydrolyzed, desalted, and lyophilized as described above. For the LC-MS/MS analysis pre-experiment, 1 μg of the mixture was eluted for use in a “label-free” method with an EASY-nLCTM 1200 UHPLC system (Thermo Scientific, Waltham, MA, USA) coupled with a Q Exactive series mass spectrometer (Thermo Scientific, Waltham, MA, USA). Raw data were searched using PD 2.2 software in full scan mode with a sequential PRM pattern. Peptides were selected using Skyline software based on reproducibility and stability. In formal LC-MS/MS experiments, equivalent peptides pretreated with trypsin were spiked with the same amounts of isotope-labeled peptides as the internal standard. The samples were analyzed using full-scan and a PRM pattern as described above. For offline data analysis, the peak area of each target protein was corrected according to the internal standard peptide to make it available for the subsequent evaluation of relative abundance.

## 3. Results

### 3.1. Daily Milk Yields and Litter Sizes Alive

**Figure 1:**
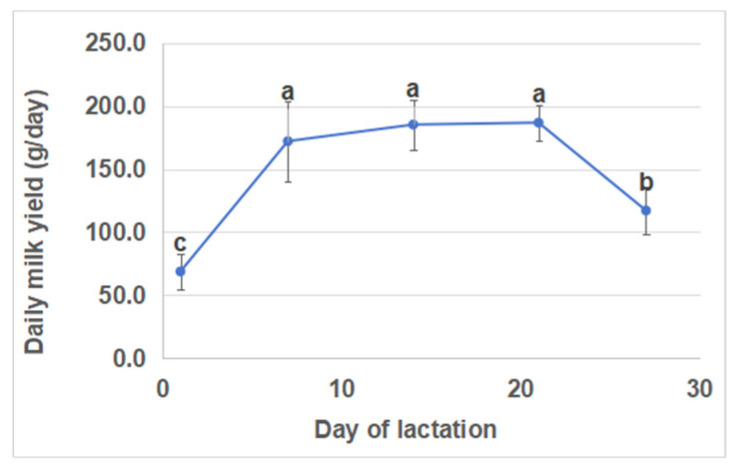
Daily milk yield at five days of lactation. Different lowercase letters indicate significant differences (*p* < 0.05), while the same lowercase letters indicate no significant difference (*p* > 0.05).

**Figure 2:**
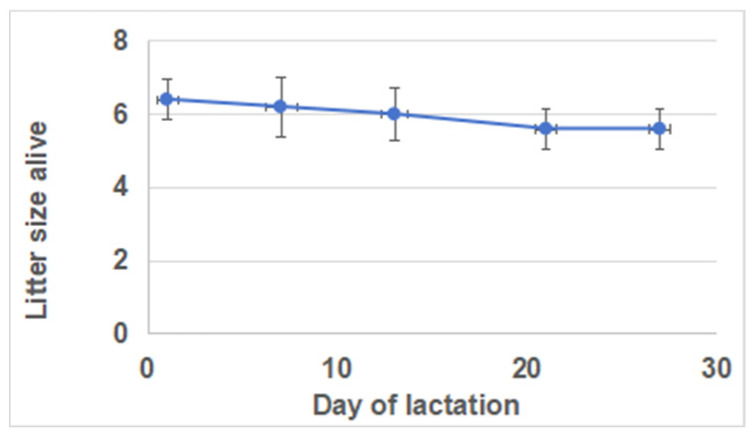
Litter size (kits born alive) at the 1 d, 7 d, 14 d, 21 d, and 28 d during lactation.

The daily milk yields were lowest (68.8 ± 13.8 g) on day 1 and highest (186.8 ± 14.1 g) on day 21 of lactation (Figure 1). Compared with days 1 and 28, there was a significant increase in milk yields on days 7, 14, and 21 of lactation. However, there were no significant differences in milk yields between days 7, 14, and 21. Furthermore, there were no significant differences in litter sizes (kits born alive) across different lactation periods (Figure 2).

### 3.2. Component Analysis of Proteins from Colostrum and Mature Milk

**Figure 3:**
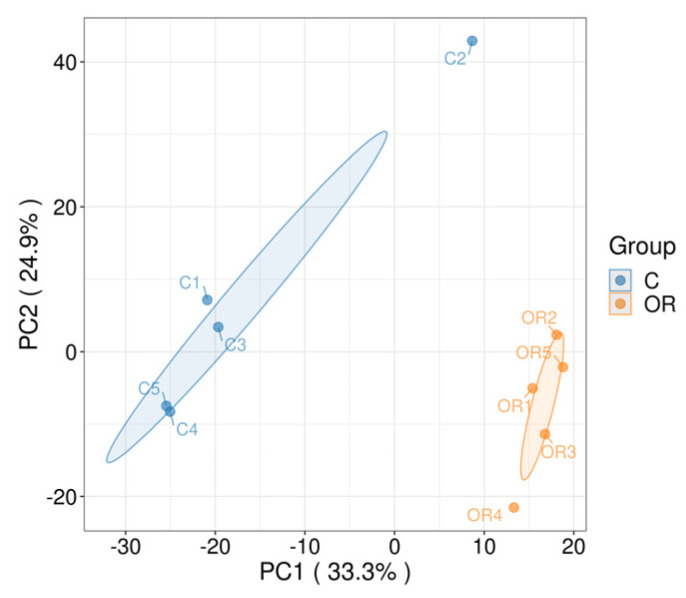
PCA plot of rabbit colostrum and mature milk. C: colostrum, OR: mature milk.

A total of 2011 protein groups and 9814 peptides were quantitatively identified in each raw milk sample using the DIA-based proteomic technique, with an FDR of 1%. Next, detection stability and sample groupings were assessed. Pearson’s correlation analysis and PCA results are shown in Figure 3. According to the score plots, the protein profiles of colostrum and ordinary milk components were clustered, with these two groups clearly divided and showing good grouping features.

### 3.3. Characterization of DAPs

**Figure 4:**
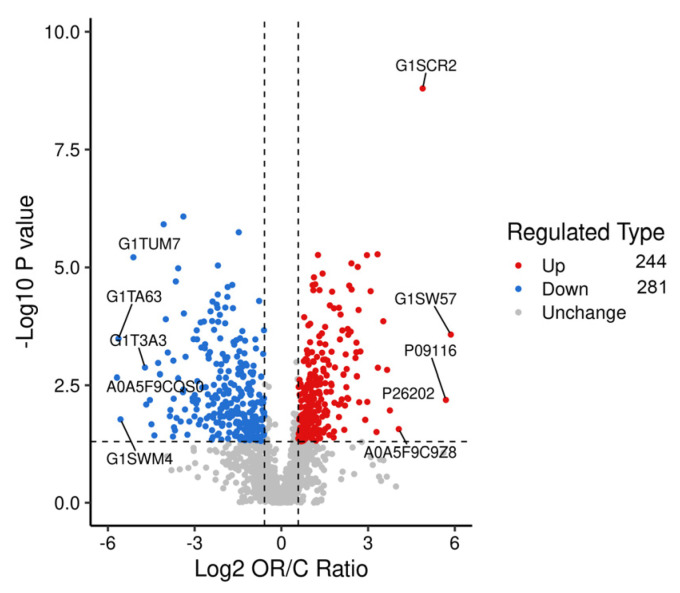
Volcano diagram of differential proteins in colostrum and mature milk.

**Figure 5:**
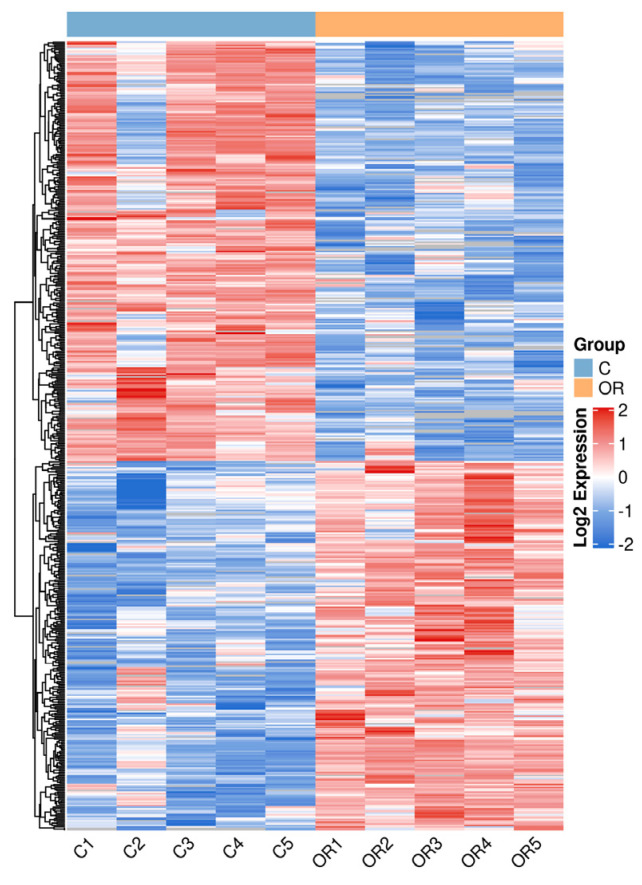
Differential protein abundance pattern clustering heat map. C: colostrum, OR: mature milk.

Proteins in each group were compared, and proteins with fold changes > 1.5 or <0.67 and *p*-values < 0.05 were considered DAPs. A total of 525 DAPs, 244 upregulated and 281 downregulated, were identified (Figure 4 and Table S1). The main upregulated DAPs were 5′-nucleotidase (NT5E), beta-casein (CSN2), protein C receptor (PROCR), neuropilin (NRP1), and fatty acid synthase (FASN). The downregulated DAPs mainly included peptidase inhibitor 15 (PI15), catenin beta 1 (CTNNB1), chitinase 3-like 2 (CHI3L2), serpin family B member 11 (SERPINB11), and transforming growth factor beta-3 proprotein (TGFB3). The overall similarity of all DAP patterns was hierarchically clustered (Figure 5). The results demonstrated a significant distinction between the samples of colostrum and mature milk, with samples within each group exhibiting strong clustering and high consistency.

### 3.4. GO Annotation and KEGG Pathway Analyses

**Figure 6:**
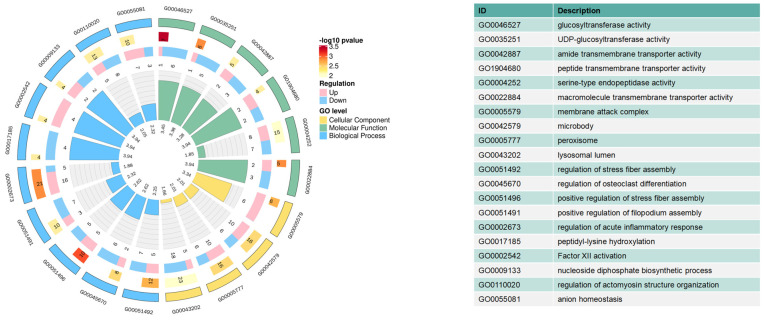
GO terms based on DAPs in the colostrum and mature milk. The first lap indicates the top 20 terms, categorized by different colors. The second lap indicates the number of DAPs and their *p*-value. The third lap indicates the quantity of upregulated and downregulated DAPs. The fourth lap indicates the fold enrichment level for each function after log2 transformation.

**Figure 7:**
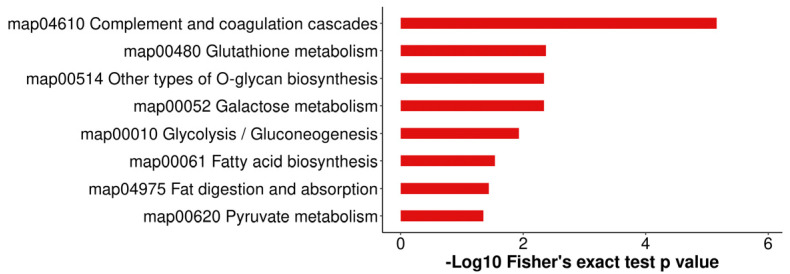
KEGG annotation of DAPs in the comparison of colostrum and mature milk. The length of bars indicates the number of proteins annotated in each pathway. The *p*-value is marked at the end of each bar.

All identified proteins were annotated to GO functions (Figure 6 and Table S2). The GO enrichment analysis confirmed the involvement of 30 significant GO terms, including glycosyltransferase activity, macromolecule transmembrane transporter activity, and regulation of acute inflammatory response. In the present study, 309 of the 525 identified DEPs were subjected to KEGG pathway analysis. Eight pathways were significantly enriched (*p* < 0.05), as shown in Figure 7 and Table S3. Complement and coagulation cascades were the most significant pathways, followed by glutathione metabolism, galactose metabolism, other types of O-glycan biosynthesis, glycolysis/gluconeogenesis, fatty acid biosynthesis, fat digestion and absorption, and pyruvate metabolism pathways.

### 3.5. PPI Network and Module Analysis

**Figure 8:**
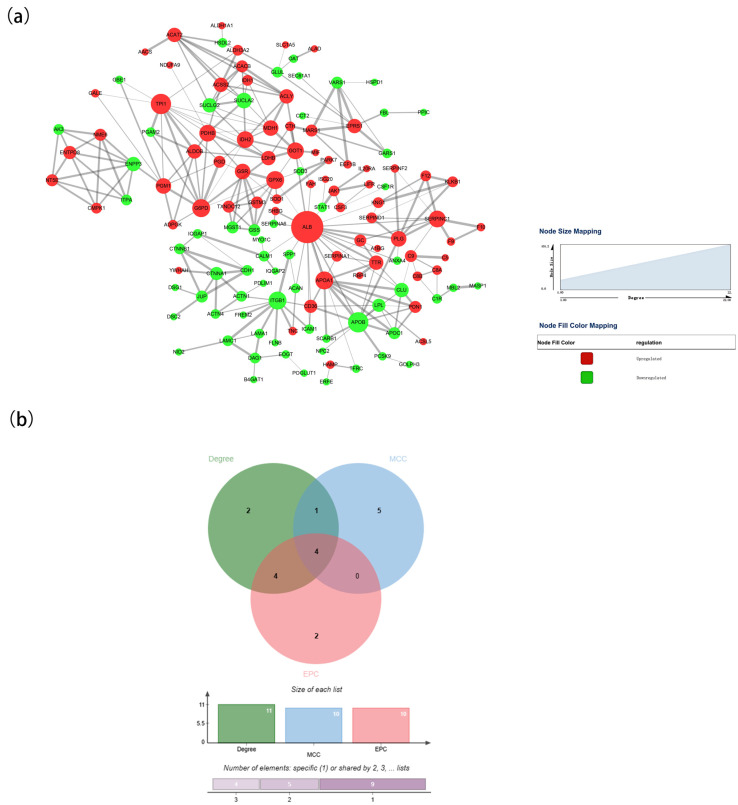
Interaction analysis of DAPs. (**a**) PPI networks of DAPs in the colostrum and mature milk groups. (**b**) Venn plot identifying significant hub proteins.

Using the STRING database and Cytoscape software (version 3.10.0), we constructed a PPI network of the DAPs identified in rabbit colostrum and mature milk. As shown in Figure 8a, the PPI network included 134 nodes and 265 edges. Among the 134 nodes, the top 10 proteins in the PPI network were evaluated using three centrality methods (Degree, EPC, and MCC) in the CytoHubba plugin. Moreover, the intersections of these three algorithms were obtained, and a Venn plot was generated to identify hub proteins (jvenn (inra.fr)) (Figure 8b). Apolipoprotein B (APOB), apolipoprotein A1 (APOA1), triose phosphate isomerase 1 (TPI1), and albumin (ALB) represented nodes with the highest degrees of biological regulation in the colostrum and mature milk groups.

### 3.6. Validation of DAPs by PRM

**Figure 9:**
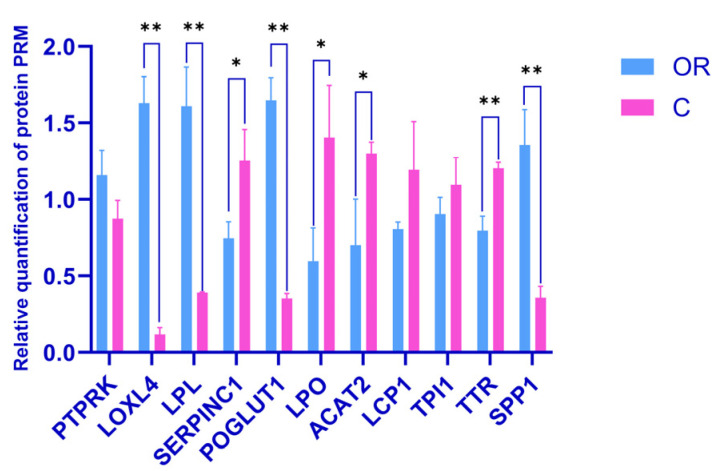
Verification of the DAPs by PRM. C: colostrum, OR: mature milk. * and ** represent *p* < 0.05 and *p* < 0.01, respectively.

A PRM assay was used to confirm the identification of several DAPs by DIA analysis. Based on proteins identifications with two or more unique peptides, a 10-sample (3-replicate) cohort derived from the two groups was sent for PRM assay to verify the relative expression. Eleven proteins from the DIA proteomic analyses, including downregulated PTPRK, LOXL4, LPL, POGLUT1, and SPP1, and upregulated SERPINC1, LPO, ACAT2, LCP1, TPI1, and TTR, were selected for the analysis. The relative expression levels of these proteins are shown in Figure 9, Table S4. The expression trends of all selected DAPs were consistent with those from the DIA results.

## 4. Discussion

### 4.1. Daily Milk Production and Associated Traits

Litter growth and mortality rates during the suckling period are highly dependent on the doe milk, and the early livability and growth performances are closely related to the quantity and quality of the milk ingested [23,24]. The milk yield of the dam plays a pivotal role in the growth of the kits during the lactation period. Studies on lactation curves have indicated a gradual increase in milk yield from parturition until the end of the third week, reaching its peak daily yield in rabbits bred for meat, followed by a subsequent decline [25]. However, the maternal Hycole line exhibited a peak in daily milk production on day 17 of the lactation period, which remained consistently high until day 21, with a slight subsequent decrease [7]. The present study analyzed the daily milk yield during five lactation stages. The highest level was observed on day 21, which is consistent with other research results involving German Angora rabbits [26]. Additionally, it has been reported that the number of kits has an impact on daily milk production [7,27]. The present study demonstrated that the litter size at different lactation stages was similar (*p* > 0.05) during the entire lactation period, and that the number of kits did not exert any influence on milk production.

### 4.2. Screening of Candidate Proteins in Colostrum and Mature Milk

To date, studies on rabbit milk have been limited. Previous studies have confirmed that the chemical composition of rabbit colostrum, including total solids, protein, and fat content, is higher than that of mature milk [7]. Furthermore, research has shown that the composition of rabbit milk is not fixed and changes significantly through different lactation stages [12]. However, no studies have been conducted to determine differentially expressed low-mass protein profiles in rabbit milk. DIA is a high-profile mass spectrometry acquisition technology developed in recent years that has led to new discoveries in quantitative proteomics. The biggest advantage of DIA over other proteomic technologies is its efficient determination of relatively low-abundance proteins in complex samples, which greatly improves the reliability of quantitative analyses [28]. Casein is the major protein in the milk of mammals. It influences a wide range of nutritional, functional and biological activities [29]. In this study, caseins, including CSN2 and Alpha-S2-casein (CSN1S2), were significantly more highly expressed in mature milk, a finding similar to that reported by Ribeiro et al. [30], for different lactation phases of human milk. Notably, upregulated proteins such as CSN2, CSN1S2, NT5E, and FASN have been reported to be associated with a significant positive correlation to milk yield [31,32,33]. Our findings on daily milk production further support this observation in line with previous reports. Furthermore, we found that several proteins, such as CHI2L2 and TGFB3, participate in immune response and are highly expressed in colostrum. CHI2L2 is one of the chitinase-like proteins (CLPs), which belong to the glycoside hydrolase 18 family [34]. It has been previously reported that CHI2L2 may serve as a potential biomarker for disease resistance in cattle breeds [35]. TGFB3, a member of the TGF-beta superfamily, may play an important role in infant immune maturation, and its abundance in colostrum is significantly higher than that in mature milk as reported in studies on human milk [36]. Because of the complex protein composition and potent bioactivities of rabbit colostrum and mature milk, characterizing as many differential changes as possible can help us understand changes in associated regulatory pathways.

The GO terms that exhibited the most significant enrichment were associated with glucosyltransferase activity. Previous studies have demonstrated the crucial role of glycosyltransferases in glycan synthesis [37]. In human and bovine milk, the concerted action of various glycosyltransferases is required for free oligosaccharide synthesis [38]. Biosynthetic steps for oligosaccharides in the mammary gland remain largely unknown; however, proteins that regulate the activity of glycosyltransferases, glycosidases, and sugar transporters play a role. However, further research is required to confirm this hypothesis and uncover the underlying mechanism.

Functional analysis based on KEGG pathways indicated that complement and coagulation cascades were those most significantly associated with DAPs. These pathways are critical parts of the innate immune defense against pathogens and play an important role in maintaining balance in the coagulation–fibrinolytic system [39], both of which are critical for infant health [40]. Interestingly, the crucial roles of the complement and coagulation cascades observed in rabbit colostrum and mature milk agreed with previous results from the milk proteomes of humans, bovines [41], goats [15,42], and yaks [43]. The fatty acid biosynthesis pathway was also significantly enriched. Fatty acid biosynthesis plays a significant role in the growth and survival of diverse organisms [44]. Fatty acids in rabbit milk are derived from blood triglycerides and synthesized de novo in the mammary glands [3]. Parturition is characterized by metabolic transitions that modulate the fatty acid profiles of colostrum and mature milk. The colostrum is rich in lipid molecules essential for newborns, including elevated concentrations of n-3 polyunsaturated fatty acids (PUFA), palmitic acid (C16:0), phospholipids, and cholesterol [45]. From a teleological perspective, higher concentrations and yields of specific fatty acids in the colostrum than in transition and mature milk may indicate a biological need in neonatal rabbits. Similar results have been reported in donkey milk. The DAPs between the mature milk (30–180 d) and colostrum (1d) of donkeys were mainly involved in fatty acid biosynthesis, fatty acid metabolism and adipocytokine signaling pathway. The related proteins ensure the healthy development of a donkey foal’s intestinal tract and facilitate the efficient absorption and utilization of nutrients [46]. Therefore, the identified DAPs participate in several important pathways that perform critical functions. Thus, our study provides novel insights into DAPs in rabbit milk across different lactation stages and reveals associated KEGG pathway differences. These results provide new information on the biological functions of rabbit milk proteins.

Based on the PPI network of DAPs, we identified four hub proteins: APOB, APOA1, TPI1, and ALB. APOB is the primary lipoprotein in chylomicrons, with very low-density. It plays a pivotal role in the formation and stability of lipoproteins and is essential for lipid and cholesterol transport to peripheral tissues [47]. Furthermore, APOB has been reported to affect hepatic lipid metabolism, steroid biosynthesis, and cell membrane functions, with potential effects on fertility, growth, and health [48]. In ruminants, this protein is associated with milk, fat, and protein yields [49,50]. APOA1 protein is a major component of high-density lipoproteins that participate in the regulation of reverse cholesterol transport [51]. APOA1 has been linked to triglyceride-enriched lipoprotein metabolism, host defense against pathogens, inflammation, and cell structure maintenance [52,53]. TPI1 catalyzes the interconversion of dihydroxyacetone phosphate and glyceraldehyde 3-phosphate during glycolysis and gluconeogenesis, respectively. This enzyme plays an important role in several metabolic pathways and is essential for efficient energy production [54]. Finally, ALB is a widely studied protein that is highly expressed in the whey proteins of many species. Most ALB proteins are associated with the immune, endocrine, transport, and catabolic systems [55]. The health, mortality, and morbidity rates of neonatal ruminants depend on colostrum quality and immunoglobulin G (IgG) absorption, and a previous study demonstrated a negative correlation between IgG concentration and ALB expression [56]. Overall, the results of the PPI network analyses of DAPs provide new insights into the functions of proteins in the colostrum and mature milk of rabbits, which reflects the specific needs of kits during different lactation stages.

## 5. Conclusions

In conclusion, our study revealed that the highest level of daily milk production was observed on day 21 during lactation, while the litter size (of kits born alive) remained consistent across different lactation stages and was not influenced by daily milk production. We identified 2011 proteins in the colostrum and mature milk of rabbits using DIA quantitative proteomics. Of these, 525 were DAPs. Regulation of the acute inflammatory response (GO annotations) and complement and coagulation cascades (KEGG pathways) were the most significant biological functions and pathways of DAPs. Based on the PPI analysis, APOB, APOA1, TPI1, and ALB were identified as the hub proteins responsible for differences in biological properties in rabbit colostrum and mature milk. The present study enhances our comprehension of the rabbit milk proteome and contributes to the expansion of knowledge regarding the requirements of neonatal rabbits. These findings provide insights for the further exploration of rabbit milk and establish a solid theoretical foundation for the optimization and utilization of colostrum products.

## Data Availability

The data are available from the corresponding author upon reasonable request.

## Supplementary Materials

The following supporting information can be downloaded at: https://www.mdpi.com/article/10.3390/biology13080634/s1, Table S1: Differentially abundant proteins in the colostrum and mature milk of rabbits; Table S2: Significantly enriched GO terms based on DAPs in the colostrum and mature milk; Table S3: Significantly enriched KEGG pathway results based on DAPs in the colostrum and mature milk; Table S4: Relative quantification of differential protein PRM.

## Abbreviations

DIA: Data-Independent Acquisition; DDA: Data-Dependent Acquisition; LC–MS: Liquid Chromatograph–Mass Spectrometer; DAPs: Differentially Abundant Proteins; PCA: Principal Component Analysis; GO: Gene Ontology; KEGG: Kyoto Encyclopedia of Genes and Genomes; PRM: Parallel Reaction Monitoring; UHPLC: Ultra-High Performance Liquid Chromatography.
